# Plasma Ceramides Pathophysiology, Measurements, Challenges, and Opportunities

**DOI:** 10.3390/metabo11110719

**Published:** 2021-10-21

**Authors:** Melania Gaggini, Alessandro Pingitore, Cristina Vassalle

**Affiliations:** 1 Institute of Clinical Physiology, National Research Council, 56124 Pisa, Italy; mgaggini@ifc.cnr.it (M.G.); pingi@ifc.cnr.it (A.P.); 2 Fondazione CNR-Regione Toscana G. Monasterio, Via Moruzzi, 1, 56124 Pisa, Italy

**Keywords:** ceramides, aerobic exercise, cardiometabolic risk and disease, lipidomics

## Abstract

Ceramides are a family of lipid molecules, composed of sphingosine and a fatty acid, and transported by lipoproteins (primarily by low-density lipoproteins) in the bloodstream. They are not only structural lipids, but multifunctional and bioactive molecules with key roles in many important cellular pathways, such as inflammatory processes and apoptosis, representing potential biomarkers of cardiometabolic diseases as well as pharmacological targets. Recent data reported ceramide modulation by diet and aerobic exercise, suggesting nutrients and exercise-targeting sphingolipid pathways as a countermeasure, also in combination with other therapies, for risk and progression of chronic disease prevention and health maintenance. In this review, we focus on the available data regarding remarks on ceramide structure and metabolism, their pathophysiologic roles, and the effect of dietary habit and aerobic exercise on ceramide levels. Moreover, advancements and limitations of lipidomic techniques and simplification attempts to overcome difficulties of interpretation and to facilitate practical applications, such as the proposal of scores, are also discussed.

## 1. Introduction

Ceramides are a family of lipid molecules, composed of sphingosine and a fatty acid, and transported by lipoproteins (primarily by low-density lipoproteins) in the bloodstream. They are not only structural lipids, but act as multifunctional and bioactive molecules in many important cellular pathways (e.g., inflammatory processes and apoptosis) [1].

For this reason, ceramides have been found to be associated with many pathological states. In particular, a close relationship has been found between elevated ceramides and type 2 diabetes (T2D) [2]. Moreover, distinct serum ceramides and other sphingolipids may predict incidence of T2D years before the onset of the disease, with this association that seems largely mediated through β-cell dysfunction [3]. Circulating ceramides may also predict cardiovascular disease (CVD) (e.g., coronary artery disease-levels of CAD, stroke, heart failure, and atrial fibrillation) [3]. Recent studies have later expanded the role of ceramides to other conditions, such as neurodegenerative diseases and cancer [4,5,6]. Interestingly, in the complex relationship between ceramides, T2D and CVD, exercise, and cognitive functions, recent data demonstrated that specific ceramide species associated with T2D in patients with CAD predicted poorer cognitive responses (e.g., less improvement in verbal memory, and less visuospatial memory improvement) to exercise [7]. Moreover, at baseline, T2D-specific ceramide species were associated with poorer memory, attention, and psychomotor processing speed performance [7]. Thus, these results recognized the complex network between chronic degenerative diseases and demonstrated how these sphingolipid pathways might represent targets of pharmacological (e.g., metformin) or exercise-interventional strategies to protect against or restore cognitive decline in CAD patients with T2D.

Ceramides may be modulated by a broad range of other determinants, including hormonal stimuli (e.g., progesterone, nerve growth factor), pro-inflammatory mediators (tumour necrosis factor, interferons, endotoxins, LPS), pharmacological treatment (e.g., metformin), ultraviolet irradiation or ionizing radiation, and lifestyle habits (e.g., Mediterranean diet and exercise) [8,9,10,11]. In particular, whether it is well known that exercise is beneficial on the traditional lipid profile (both in healthy subjects than in cardiometabolic patients), its effects on ceramides need to be better studied [12,13,14].

In recent years, lipidomics, improved by the innovations in mass spectrometry (MS) and chromatographic technologies, permits better identification of the composition of lipid molecular species in biological samples [15]. With these advancements, increasing data have demonstrated that aerobic exercise improves ceramide profile in skeletal muscle and in the bloodstream [16,17]. Moreover, chronic exercise reduces ceramide levels in subjects with obesity or T2D [18].

However, techniques of ceramide analysis are not so widespread in routine laboratories and interpretation and practical application of ceramide results in the clinical practise could be difficult for many physicians. In this context, there are efforts to render these biomarkers more usable, with the proposal and evaluation of friendlier circulating ceramide risk scores (e.g., CERT 1, CERT 2, SIC score, dSCORE) [15,16,17,18,19].

In this review, we focus on the available data regarding remarks on ceramide structure and metabolism, the modulation of ceramides by aerobic exercise, and pathophysiologic significance of this relationship. Moreover, advancements and limitations of lipidomic techniques and simplification attempts of translation of results to overcome difficulties of interpretation and to facilitate practical applications are also discussed.

## 2. Ceramide Chemical Structure and Biological Activity

### 2.1. Chemical Structure and Production

**Figure 1:**
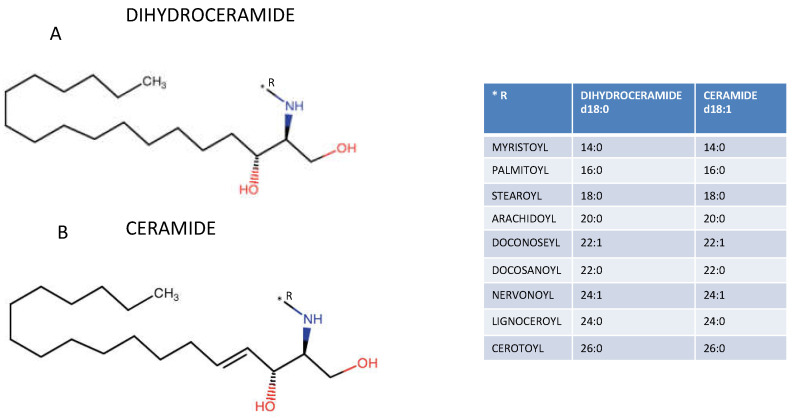
(**A**) Dihydroceramide structure (**B**) Ceramide structure. The table reports the different fatty acids with variable length joined by an amide linkage.

The chemical structure of ceramides consists of a sphingoid base (e.g., dihydrosphingosine, sphingosine, phytosphingosine or 6-hydroxy sphingosine), which is a long-chain amino alcohol, and one among several fatty acids, different in chain length and degree of saturation, joined by an amide linkage (Figure 1) [20]. The addition of polar groups results in sphingolipid classes that vary in their underlying ceramide, whereas the addition of variable sugar groups results in different glycosphingolipids, adding a further level of complexity and heterogeneity [20].

**Figure 2:**
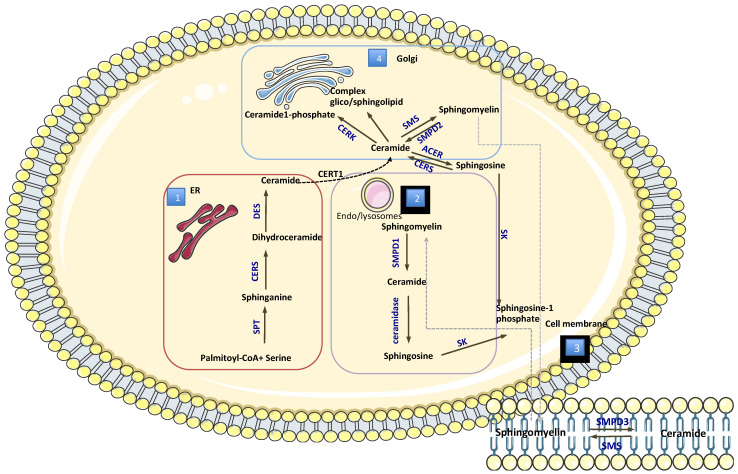
1: De novo synthesis occur in endoplasmic reticulum (ER) by the activation several enzymes such as SPT: Serine Palmitoyltransferase; CERS: Ceramide synthases; DES: Dihydroceramide desaturase. 2: Salvage pathway is the synthesis of ceramide from sphingosine that occur in endo/lysosomes by the action of SMPD1: Sphingomyelinase 1 and ceramidase. 3: catabolic pathway occur on the cellular membrane by the hydrolysis of ceramides in the presence of Sphingomyelin synthases (SMS) sphingomyelin is formed, by Sphingomyelinase 3(SMPD3), ceramides is formed. 4: Transposrt and metabolism occur in Golgi apparatus. CERK: ceramide kinase; SMPD2: Sphingomyelinase2; SMS:Sphingomyelin synthases; CERT1: ceramide transfer protein; ACER: alkaline/neutral ceramidase; SK sphingosine kinase.

There are three major pathways by which ceramides are synthesized (Figure 2) [21,22]:

*Sphingomyelinase pathway*: this is a catabolic pathway, considered a “fast” way to generate ceramides, that involves enzymes which hydrolyze sphingomyelin in the cell membrane producing ceramides and phosphocholine.

*The de novo pathway*: generating ceramide from less complex molecules, this pathway occurs in the endoplasmic reticulum, where serine palmitoyltransferase catalyzes the condensation of palmitate and serine to form 3-keto-dihydrosphingosine (the rate-limiting step of the pathway). Then, 3-keto-dihydrosphingosine reductase produces sphinganine, and through the action of different isoforms of ceramide synthase, which promote the reaction between of acyl-CoA of different chain lengths to the molecule of sphinganine, dihydroceramide are produced. The last step is taken by dihydroceramide desaturase, which introduces a double bond in position 4–5 trans of dihydroceramide to form ceramides. Ceramides are then transported to the Golgi apparatus, where they can be further metabolized to other sphingolipids (e.g., sphingomyelin).

*Salvage pathway*: these occur in endo-lysosomes providing that sphingomyelin that are broken down into sphingosine through the action of ceramide synthase are reused to be converted into ceramides.

In addition to these main pathways, ceramides may be transported to the Golgi apparatus and metabolized to other sphingolipid groups.

### 2.2. Ceramides as Structural Elements of Biological Membranes as Well as Bioactive Molecules

Together with cholesterol, sphingolipids also represent the basic lipid component of lipid rafts, cholesterol- and sphingolipid-enriched membrane domains in contiguity with the external leaflet of the plasma membrane [23].

Concentration of sphingomyelin in the lipid rafts is higher than in non-raft portions of the membrane, where appropriate ratios in the lipid rafts is critical to face redox injuries, such as for example those caused by radicals such as nitric oxide-•NO [24].

Lipid rafts are involved in the modulation of multiple signal transduction pathways beginning at the cellular membrane, which involve, but are not limited to activation of PI3K and Akt kinases (PI3K-PKB/Akt pathway, modulating cell metabolism, growth, cellular proliferation, and survival), clustering of Fas/CD95 (leading to apoptosis, activation of caspase cascades, and ceramide generation), activation of erythropoietin, and immune signaling (e.g., B- and T-cell receptor signal activation) [25,26,27,28].

However, further studies are needed to better understand the specific contribution of the subclasses of sphingolipids and ceramides in lipid raft formation and functioning.

Originally viewed only as structural lipids of the cellular membranes, ceramides have been primarily related through multiple mechanisms to cell death (e.g., by oxidative stress, increased auto-mitophagy, and mitochondrial dysfunction) and apoptosis (e.g., by modulation of phosphatase PP2A and cathepsin D [29], as well as forming pores directly in the outer mitochondrial membrane, especially involving the pro-apoptotic protein Bax), then as bioactive modulators of several intracellular functions with a role in many pathophysiological pathways (e.g., inflammation, apoptosis, cell growth arrest, differentiation, cell senescence, cell migration and adhesion) [1,29,30,31,32,33]. However, ceramide is not a single lipid; there are, in fact, several chemically different molecules that may largely vary in their chemical structure, which is reflected in different biological actions so that different species, with key roles in cellular functioning, can have different signaling properties, and even exert opposite effects in different anatomical district (Figure 1) [3]. For example, generally ceramide and sphingosine are considered pro-apoptotic, although long-chain ceramides are generally considered to be more apoptotic, whereas very-long-chain ceramides—e.g., C24-ceramide—are more pro-survival, since the equilibrium between long- and very-long-chain ceramides is probably essential to determine the fate of the cell, whereas sphingosine-1-phosphate is considered a pro-survival molecule [34,35,36,37,38,39]. Additionally, dihydroceramides, in the past considered inactive precursors to the bioactive sphingolipids, exert several biological actions, including enhancing autophagy and the unfolded protein response (intracellular stress in response to the accumulation of unfolded or misfolded protein in the endoplasmic reticulum), and reducing cell proliferation [40,41].

For their importance in key cellular pathways, ceramides and their precursor or downstream metabolites have been involved in several pathophysiological conditions such as cancer, neurodegeneration, and cardiometabolic diseases [4,5,42,43,44,45]. In particular, circulating ceramides are elevated in obese subjects, prediabetes, and type 2 diabetes, and correlated with the risk of developing atherosclerosis and cardiovascular disease [46].

## 3. Modulation of Ceramides by Diet and Endurance Exercise

In the PREDIMED study (Prevención con Dieta Mediterránea), the ceramide score, which was calculated as a combination of four ceramides (C16:0, C22:0, C24:0, and C24:1), was associated with a 2.18-fold higher risk of CVD, with traditional Mediterranean diet enriched with extra-virgin olive oil or nuts that retains the potential to mitigate the adverse effects of elevated ceramide on CVD risk [10]. Additionally, the Nordic diet (characterized by foods such as whole grains, fruit, berries, root vegetables, cabbage, fish, and rapeseed oil) appeared able to reduce insulin-resistance-inducing ceramides after 12 weeks [47]. All together these results suggested the potential to favorably modulate ceramides with adherence to a healthy dietary habit.

Exercise may modulate the muscle total content of ceramide fatty acids, changing their composition in skeletal muscle, specifically reducing ceramide content and improving muscle glucose metabolism as a reflex of ceramide involvement in regulation of glucose uptake by muscle [48,49,50]. Specifically, an increase in ceramide concentration leads to insulin-resistance promotion (e.g., activating intracellular inflammatory pathways and increasing cytokine production by macrophage through Toll-like receptor 4 signaling), whereas a decrease in ceramides is associated with increased insulin sensitivity, this trend accounting, at least partly, to the beneficial exercise effects [51,52]. One reason for the molecular link between ceramides and insulin may involve the excess saturated free fatty acids-FFA, through the inhibition of Akt/PKB phosphorylation (a serine/threonine kinase involved in insulin-stimulated anabolic metabolism), thus preventing the translocation of Akt/PKB from the cytoplasm to the membrane, and leading to impaired insulin signaling [53].

Baranowski et al. demonstrated that exercise increased levels of sphingoid base-1-phosphate (sphingosine-1-phosphate-S1P and sphinganine-1-phosphate-SA1P-terminal breakdown product of ceramide and an important anti-inflammatory and vascular signaling lipid mediator associated with lipoproteins, particularly high-density cholesterol-HDL) in rat skeletal and cardiac muscle [54,55]. Moreover, the same authors reported apparently controversial results in humans, as they found that that 60 min cycling exercise at 70% of maximal oxygen consumption (VO_2 max_) increased plasma levels of S1P and SA1P and decreases erythrocyte ceramide levels more in untrained than in endurance-trained subjects, whereas a 48-hour ultramarathon run markedly reduced circulating concentration of these sphingoid base-1-phosphates [56,57]. In a subsequent study, the same group demonstrated that these differences may be explained by variation in the release of sphingosines that follows a time- and intensity-dependent way according to type of exercise. Specifically, authors found that exercise until exhaustion increased plasma concentration of S1P and SA1P, whereas moderate-intensity exercise elevated only SA1P level, so that muscle S1P content is increased in proportion to exercise duration and intensity, whereas plasma S1P level is elevated only by very high-intensity exercise [58].

Importantly for data interpretation, it must be taken into account that sphingolipids and ceramides are produced by various cells and tissue districts, levels of ceramides measured in blood may reflect different contributions from multiple origins, other than skeletal muscle.

Many other findings suggested that ceramide levels in the muscle are reduced following exercise training [50,59]. Specifically, exercise has been shown to decrease intramyocellular ceramide levels in a small sample of eight participants, who underwent submaximal intensity walking or cycling training (50–70% VO_2 max_, 4–5 times per week, 45 min each session) for 16 weeks [49].

Experimental data suggested that high-fat diet-induced obesity is accompanied by an increase of ceramides, activation of inflammasome, and a release of cytokines (e.g., IL-18) [60]. This condition appears improved by both endurance (ET-treadmill at 80% VO 2 max for 30 min/day, 5 times/week for 10 weeks) and resistance (RT-mice gripped with their front and their back paws on a horizontal wire of the metal mesh placed in a vertical position [60]).

Strength training performed for 5 times/week, 3 min, 3 series of training, decreased body weight (*p* < 0.05) and reduced serum ceramides (*p* < 0.005) and inflammatory cytokine levels, improving glucose tolerance (*p* < 0.001), with ET being more effective than RT [60].

Contrary to the expectation that physical activity lower ceramide by improving lipid oxidation capacity, a combination of dietary supplementation (nutrient bar) plus exercise significant increased ceramides (e.g., C14:0, C16:0, C20:0 and C22:0) [61]. Other data similarly demonstrated that acute exercise transiently increases serum ceramides in obese untrained subjects [49]. In these cases, the observed elevation in ceramides may therefore reflect transient increases in fatty acid mobilization, whereas regular training, resulting in an increased muscle fatty acid oxidation capacity, may normalize this pattern.

Accordingly, circulating ceramide levels can be reduced through physical training in patients with type 2 diabetes. In particular, 12 weeks of aerobic exercise training in obese or diabetic subjects (60% of their maximal heart rate-HR_max_; ~50% VO_2max_, gradually increasing intensity so that by fourth week 80–85% of HR_max_ ~70% VO_2max_ was reached) and 16 weeks of exercise (4–5 days/week, 45 min/session of moderate intensity determined by heart rate, stationary cycling or walking) in overweight/obese subjects may reduce plasma sphingolipids (e.g., C14:0, C16:0, C18:1, and C24:0) [18,46].

When basal relationships and the effect of acute exercise (1.5 h at 50% VO_2max_) and recovery of serum ceramide and sphingolipid content in sedentary obese individuals, endurance-trained athletes (mixed group including cyclists, triathletes, and runners), and individuals with type 2 diabetes (T2D) was evaluated, basal serum C18:0, C20:0, and C24:1 ceramides and total dihydroceramide were significantly higher in T2D [46]. Although no differences in serum ceramides and sphingolipids were observed between endurance-trained athletes compared with obese individuals, suggesting minimal changes with chronic exercise training, basal serum C18:0 was significantly lower in athletes than in T2D patients. This finding is noteworthy, as C18:0 is particularly adverse for insulin sensitivity in humans, thus it could act as a reliable biomarker in this context [62]. Acute exercise significantly increased serum ceramide, which decreased to basal values during recovery, with C16:0 and C18:0 ceramide and C18:0 sphingomyelin positively correlated with inflammatory muscle markers, and C14:0, C22:3, and C24:4, positively associated with insulin secretion and glucose tolerance, suggesting that that single ceramide and sphingolipid species drive metabolic protective or deleterious effects, representing possible therapeutic targets for the future [46]. In any case, at the serum sphingomyelin, elevation after acute exercise follows a decrease during the recovery phase, which could mirror a cardioprotective response promoting insulin sensitivity [46].

Interestingly, in a study exploring metabolomic indicators of healthspan, the group of ceramides, in addition to other biomarkers (e.g., triglycerides, cholesterol, and C-reactive protein) were particularly closely related to VO_2max_ indices of cardiorespiratory fitness, an important predictor of future health and disease risk [63].

We also previously observed that total ceramides, and total diacylglycerols (DAG) significantly decreased after a marathon run in trained athletes. Moreover, we found that several ceramide classes decreased after exercise, while only one of the DAG (36:3) significantly decreased. Instead, total sphingomyelin SM and specific species did not significantly change, although a trend towards decrement of levels was observed [17].

Interestingly, recent data demonstrated the relationship between wellbeing and sphingolipids, which may be modulated, at least in part, by interventional lifestyle changes (e.g., exercise and diet), which may suggest these molecules as a target to promote health also through improved blood sphingolipid profile [64,65].

## 4. Ceramides Scores as Opportunity for Practical Application

In view of their ubiquitous nature and their prompt dysregulation during disease onset and development, practical use of single specific ceramide is highly impracticable, especially also taking into account intra- and infra-subject heterogeneity and specific pathophysiological effects, sometimes opposite, retained by each ceramide. For this last aspect, an example is the relative increase in long-chain (C16) but not in very-long-chain (C24-24:1) species, which mediate insulin resistance in mice [66,67].

Many hindrances must be overcome to render this information more usable in the practical preventive and clinical settings [15].

Ceramides can be evaluated by liquid chromatography mass spectrometry LC–MS and LC–MS/MS, LC-ESI-MS/MS, gas chromatography mass spectrometry GC-MS, or fast liquid chromatography quadrupole time-of-flight mass spectrometry [15]. In clinical laboratories, LC–MS/MS use has especially grown for high specificity and sensitivity [68]. However, these techniques are not easily implemented in all clinical laboratories, because they are still expensive, and demand specialized and delicate instruments that need careful maintenance, skilled operators, and technical expertise [15]. One dosage challenge remains the extraction phase of the sample (choice of chromatography column and internal standard are critical steps), which is largely operator-dependent, as well as the possibility of ionic suppression caused by the nature of the matrix, which can be considered [15]. Therefore, there is a lack of standardization (there is still considerable heterogeneity between laboratories when measuring the same analyte), as well as of shared reference values for ceramides. In this context, data on within- and between-subjects biological variability with evaluation of confounding factors (e.g., common lifestyle factors such as diet, circadian rhythm, smoking habit, exercise, and drugs) as well as the possibility to use a different reference range according to gender and age intervals, need to be further evaluated [15].

In this context, the strategy to identify a panel including ceramide combinations, likely with other significant biomarkers (e.g., lipids or cytokines), may assist in the interpretation of results, which can often be difficult for clinicians. Accordingly, efforts have been made in this direction, such as in the development of the CERT1 risk score based on ceramide concentrations and their ratios, and other indices [69,70].

*CERT1*—This score was calculated by Cer(d18:1/16:0), Cer(d18:1/18:0), and Cer(d18:1/24:1) concentrations and their ratios to Cer(d18:1/24:0), developed and applied to a population of patients with acute and stable coronary artery disease for the prediction of mortality [69]. CERT1 values were scored as follows—2 points to those with values in the fourth quartile, 1 point to values in the third quartile, and 0 points to the first and second quartiles, with total CERT1 ranging from 0 to 12. When patients are stratified into four risk categories (low–moderate–increased–high) a correlation was observed between cardiovascular risk and increasing score [69].

Score performance was further validated in a large-scale population-based study (FINRISK 2002; *n* = 8101), confirming the correlation between increased cardiovascular events and score grades [70].

*CERT2*—a phosphatidylcholine (PC 16.0/16.0) together with one ceramide/ceramide ratio [Cer(d18:1/24:1)/Cer(d18:1/24:1)], and two ceramide/PC ratios [Cer(d18:1/16:0)/PC16:0/22:5, Cer(d18:1/18:0)/PC14:0/22:6] form this score, developed and validated in coronary artery disease cohorts for cardiovascular death prediction, and found associated with biomarkers of inflammation, myocardial necrosis, myocardial and renal dysfunction, and dyslipidemia [71,72]. Whether CERT1 gives a certain risk in patients belonging to the third and fourth quartiles, CERT2 already in the second quartile yields a risk point [19].

*Diabetes Score (dScore)*—The same group also defined this score, which consists of a ceramide ratio [Cer(d18:1/18:0)/Cer(d18:1/16:0)] to predict the risk of developing type 2 diabetes (T2D) [73]. For this score, age, sex, and BMI were also added to obtain a 10-year absolute risk (scale 0–100) of developing T2D. The subjects evaluated were then categorized into low risk defined as <5%, moderate risk 5–15% and high risk >15% probability of developing T2D in the coming 10 years [73].

*SIC score*—Of interest is the recent application of “machine learning” for the SIC score [74]. Machine learning is a type of artificial intelligence able to learn, adapt, and improve from existing data, and draw new inferences [75]. An important characteristic of this procedure over the traditional statistic tools is the possibility to make inferences at individual rather than at group level, which greatly increase its significance and potential for clinical translation. This procedure was applied in a population of patients with coronary artery diseases against control subjects, where a panel of 32 sphingolipids was screened, to identify a score of predictive biomarkers [74]. The SIC score was then proposed, which also included different minor lipids that may account as measures of flux through the ceramide biosynthesis pathway rather than the more common abundant ceramides that are generally included in the other ceramide-based scores [74]. This score also was more effective to stratify patients with coronary disease than when using conventional clinical cardiovascular biomarkers, including traditional lipids (e.g., LDL cholesterol and triglycerides) [74].

Interestingly, the effect of 5 days of controlled bed rest on circulating ceramides was evaluated in healthy subjects of both genders (opposite experimental setting respect to the evaluation of acute and chronic exercise), showing that single ceramides decreased (especially C24:0) while ceramide ratios and the CERT1 score, associated with cardiovascular risk, increased especially in the elderly, independently of changes in circulating lipoproteins [76]. Moreover, the C18:0/C24:0 ceramide ratio corresponded to lower physical performance (6-minute walk test), and metabolic biomarkers (e.g., adiponectin, and fibroblast growth factor 2) inversely correlated with ceramides [76].

**Figure 3:**
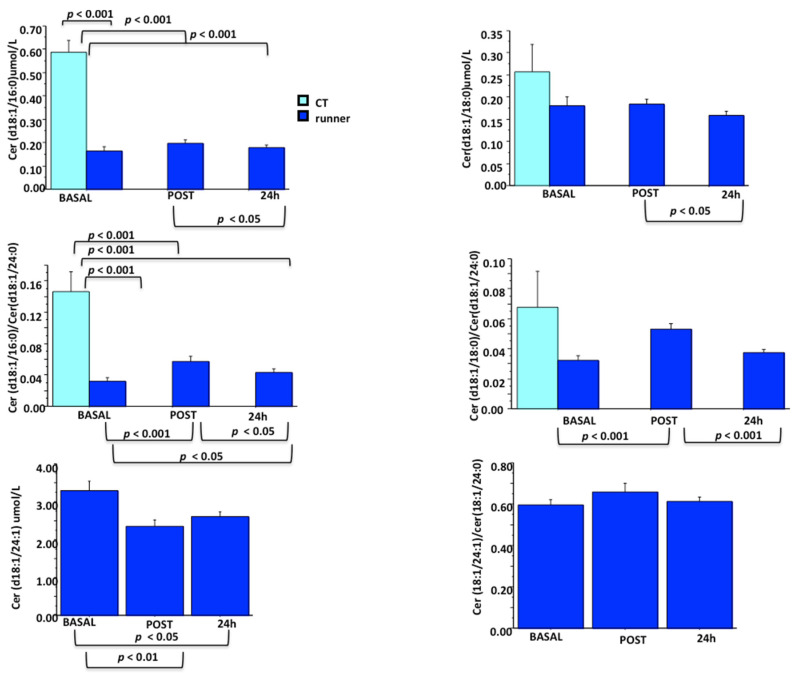
CERT1 components in sedentary subjects (*n* = 17) and in trained runners (*n* = 13) before a half-marathon and during recovery. ANOVA analysis and Scheffe’s post hoc test.

Therefore, ceramide scores appear more user-friendly than the interpretation of several single ceramides, and are promising for risk stratification in primary and secondary cardiometabolic prevention. Moreover, the possibility to apply these tools in other settings and other disease states, such as to further investigate the complex scenario of exercise pathophysiology, is not neglectable. Accordingly, we calculated some of the CERT1 score components in a population of trained athletes performing a half-marathon run, which we previously published [17], investigating the trend of ceramide, diacylglycerol, and sphingomyelin species versus a group of sedentary subjects matched for age and gender percentage (however unfortunately Cer d18:1/24:1 was not available in the control group). Results are shown in Figure 3, reporting lower levels of all ceramides and their ratio in athletes before and after the half-marathon run when compared to the sedentary subjects.

## 5. What Is in Store for the Future?

Ceramides have been widely investigated for their role as structural (e.g., formation and integrity of cellular membranes) and functional bioactive molecules (regulation of key cellular processes, e.g., inflammatory processes, and cell proliferation, and apoptosis). Nonetheless, the extreme complexity of the ceramide metabolic pathways renders their pathophysiological complex to fully understand, because an intricate network of factors may determine their resulting biological effects (e.g., number of species involved, differences in their structures, especially the length of their side acyl chain, ceramide source, and intracellular compartmentalization, changes in membrane dynamics caused by alterations in membrane sphingolipid composition, different environments as cellular and tissue types). Only a deeper comprehension of these determinants in different physiological and pathological contexts will allow possible use of these molecules as new pathogenetic determinants of disease, diagnostic biomarkers, and targets of innovative therapeutic strategies. This target will require multidisciplinary collaboration between different professionals, including laboratorists, biochemists, and clinicians developing appropriate experimental to human models. Ceramide testing implementation in the clinical practice is possible, if overcoming current pitfalls in the field. It is true that mass spectrometer instruments may be costly compared to some other analytical equipment (particularly if the reagent providers offer them at low/no price), or if they are purchased for one purpose (e.g., ceramide analytics only). On the other hand, routine clinical laboratories do have usually suitable instruments available as quite standard mass spectrometers can be used for analyses. Furthermore, labeled internal standards are currently offered for a selected set of ceramides at least by two producers, thus analyses can be carried out with high precision and absolute quantification, which is not often the case with other analytical technologies. On the other hand, even if the instruments may be considered expensive, the actual sample running costs are not and with one instrument it is possible to run hundreds of samples per day. Of note, many other laboratories are running high-volume mass spectrometer assays (e.g., hormones and vitamin D) successfully with acceptable costs. This should be possible for ceramides as well, if they can be more widely adapted to clinical use. It is true that at present analytical methods for ceramide measurement are heterogeneous and not very well comparable, which concerns mainly different research laboratories using variable methods for lipidomic analyses. However, that may not be the case with providers of tests intended for clinical use as they are likely to use same internal standards from the same source, they are likely to use similar protocols with some local adaptations, and they may also cross-check each other’s results and adjust reference values.

High circulating ceramide values have been found in obesity, diabetes, cancer, hepatic steatosis, hypertension, heart failure, and atherosclerosis, and recently reviewed studies on therapeutic drugs targeting ceramides and decreasing their levels in plasma and tissues are ongoing [2]. Results in this field may exert enormous interest for the possibility to better treat several cardiometabolic conditions, including hypertension, atherosclerosis, diabetes, and heart failure. Thus, in the near future, ceramides could be useful in cardiometabolic risk prediction as well as in the disease management, with a role over and above currently used traditional lipid biomarkers [77]. In this context, the development of shared scores (eventually mixed with other circulating, clinical or instrumental risk biomarkers) may render comprehension and use of ceramides easier in clinical practice. Moreover, dietary and aerobic training-induced modulation in the lipid profile status may identify diet and exercise-targeting sphingolipid pathways as a countermeasure, also in combination with other therapies, for the risk and progression of chronic diseases and health maintenance.
